# Pyramidal Decision Support Framework Leverages Subspecialty Expertise across Enterprise to Achieve Superior Cancer Outcomes and Personalized, Precision Care Plans

**DOI:** 10.3390/jcm11226738

**Published:** 2022-11-14

**Authors:** Linda D. Bosserman, Isa Mambetsariev, Colton Ladbury, Afsaneh Barzi, Deron Johnson, Denise Morse, Debbie Deaville, Wade Smith, Swapnil Rajurkar, Amartej Merla, George Hajjar, Daniel Kim, Jeremy Fricke, Vijay Trisal, Ravi Salgia

**Affiliations:** 1 Department of Medical Oncology and Therapeutics Research, City of Hope, Irwindale, CA 91706, USA; 2 Department of Medical Oncology and Therapeutics Research, City of Hope, Duarte, CA 91010, USA; 3 Department of Radiation Oncology, City of Hope, Duarte, CA 91010, USA; 4 Department of Clinical Informatics, City of Hope, Duarte, CA 91010, USA; 5 Department of Quality, Risk and Regulatory Management, City of Hope, Duarte, CA 91010, USA; 6 Department of Enterprise Business Intelligence, City of Hope, Irwindale, CA 91706, USA; 7 Department of Medical Oncology and Therapeutics Research, City of Hope, Newport Beach, CA 92660, USA; 8 Department of Medical Oncology and Therapeutics Research, City of Hope, Upland, CA 91784, USA; 9 Department of Medical Oncology and Therapeutics Research, City of Hope, Antelope Valley, CA 93534, USA; 10 Department of Medical Oncology and Therapeutics Research, City of Hope, Mission Hills, CA 91345, USA; 11 Department of Medicine, City of Hope, Duarte, CA 91010, USA

**Keywords:** complex case discussions, decision support, oncology pathways, personalized medicine, subspecialty expertise

## Abstract

The complexity of cancer care requires integrated and continuous support to deliver appropriate care. An expert network with complementary expertise and the capability of multidisciplinary care is an integral part of contemporary oncology care. Appropriate infrastructure is necessary to empower this network to deliver personalized precision care to their patients. Providing decision support as cancer care becomes exponentially more complex with new diagnostic and therapeutic choices remains challenging. City of Hope has developed a Pyramidal Decision Support Framework to address these challenges, which were exacerbated by the COVID pandemic, health plan restrictions, and growing geographic site diversity. Optimizing efficient and targeted decision support backed by multidisciplinary cancer expertise can improve individual patient treatment plans to achieve improved care and survival wherever patients are treated.

## 1. Introduction

The complexity of oncology care continues to increase across cancer types with discoveries of new germline and somatic mutations; new diagnostic, prognostic, and predictive testing; and new systemic, radiation, surgical and supportive therapies [1]. Luckily, this increasing complexity of diagnostic and therapeutic options can provide better outcomes for patients just as oncology care is consolidating into larger network enterprises where multidisciplinary research-focused academic oncology experts partner with their network of oncology clinicians to offer personalized precision cancer care to each patient [2,3,4,5]. For both academic and network oncologists, an increasing number of cancer patients along with the increasing complexity of diagnostics, treatments, supportive care, survival, and end-of-life care has also increased the time pressure to fully engage patients and their support systems in understanding these complexities and developing individual care plans through shared decision making [6,7].

Personalized Precision Medicine (PPM) for cancer patients means getting the correct diagnosis and therapy reviewed, ordered, and delivered for each cancer patient to achieve their best health outcomes. These personalized treatment plans depend on the patient’s disease, biomarkers, comorbidities, available trials or therapies, and personal preferences as shown by the Yale network and inclusion in the 13 components incentivized in the Center for Medicare and Medicaid’s (CMS) Oncology Care Model [8,9]. More than molecular testing is required to determine the best targeted or combined targeted or other therapies; specifically, there is a requirement for accurate, complete diagnosis and staging with biomarkers and clinical information to empower evaluations of whether the standard of care pathways, including clinical trials, are the best option or whether an individual treatment plan is better for each component of a multidisciplinary care plan [10]. The challenge for organizations is to deliver the most up-to-date diagnostic and therapeutic options to oncologists efficiently along with complex orders to safely and effectively deliver care. Multidisciplinary conferences have been shown to impact care plan changes and improve outcomes [11,12]. As the number of cancer patients seen and managed daily has increased, along with complexity, groups have come together to implement high-quality, standard-of-care pathways to cover the most common cancers [13,14,15]. Some groups have shown these pathways can improve care delivery and cancer outcomes and lower costs [16,17,18,19]. An unmet challenge is to serve patients where rapidly evolving new data on newly identified biomarkers or inherited mutations, response to prior therapies, rare histologic subtypes, rare diseases, clinical trials, and newly approved treatments make implementing an individual care plan time-consuming for busy clinicians to review and incorporate for each patient. In addition, early pathway programs sought to only cover the most common cancer presentations with the goal of 80% of those patients being targeted to be incorporated into one of their pathways [20,21,22]. However, as disease complexities have increased, enterprises need pathways to have greater depth and breadth to address known clinical settings with specific beneficial therapies. They also need processes to address rapid new information that is not yet incorporated into a formal pathway tool. Thus, new decision support frameworks are required.

**Figure 1:**
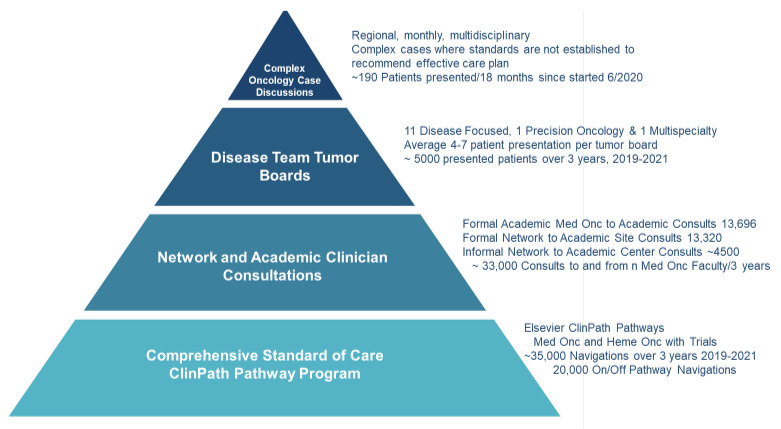
Four Components of the Pyramid of Decision Support for COH Enterprise.

City of Hope has an enterprise commitment to democratize cancer care delivery by providing expert faculty knowledge to clinicians and their patients at every network site regionally, nationally, and internationally. Challenges from the COVID-19 pandemic, health plan restrictions, and our expanding geographic network of sites led to the development of a four-tiered Pyramidal Decision Support Framework (Figure 1) to expand the superior overall survival in every cancer type and stage seen at the academic center to the enterprise’s growing network [23,24,25,26,27]. The pyramid is based on providing robust evidence-based pathways for the most common cancer presentations, enabling the availability of formal and informal faculty consultations, providing disease-specific and precision oncology tumor boards, and instituting our newest component, regional Complex Oncology Case Discussions (COCD), where multispecialty expertise is provided for patients’ presentations at a physician’s request when standards of care do not exist.

### Challenges in Development

Our pyramidal model was developed with the new COCD component in response to data demonstrating that precision oncology was adopted faster at academic centers largely due to the influence of strategic initiatives such as the NCI-MATCH trials while adoption has been slower in community practice sites [4,28]. While EGFR and ALK testing rates in NSCLC have been slowly rising in eleven reported community practice studies with ranges between 35.5–100% and 23–95%, respectively, most other alterations are still untested, and PD-L1 expression rates were reported between 1.2–56% [29,30,31,32,33,34,35,36,37,38]. These challenges are present in other cancer types in the community including in breast cancer and ovarian cancer where, genetic testing and genetic counseling has also been underutilized [39,40]. There are several primary reasons for this, including a lack of knowledge of the latest therapeutics and testing, time constraints, burdensome pre-authorizations, and the cost of precision oncology testing that requires a value-based assessment that is often missing in community practices [15,41,42,43].

Knowledge gaps in community practices have become even more challenging with the growth of immunotherapy, cellular therapies, and the availability of a growing number of targeted therapies for different cancer sub-types and lines of therapy. Failing to provide these therapies has resulted in adverse outcomes for patients treated in some community practices compared to academic centers [5,44]. The implementation of evidence-based algorithms such as ours has the potential to eliminate these knowledge gaps and improve patient outcomes. In addition, it takes time for clinicians to gain the knowledge and experience to anticipate and managing complex and unique toxicities for so many new therapies, especially for less commonly seen cancer presentations. One study reported that almost 61% of patients in the community did not complete their immunotherapy, with the leading cause being the timely management of novel or rare immune-related adverse effects (irAEs) [45]. The implementation of our algorithm directly addresses this issue by including experts in immunotherapy treatment and experts from multiple disease types who may have experience with rarer or less common irAEs in tumor boards, 1:1 faculty consultations, and COCDs. When disease leads identify new regimens or drugs for adding to our EPIC Beacon orders, they also add management information for toxicities to help network clinicians expand awareness of the timing and types of toxicities as well as their management. Furthermore, our decision support pyramid often identifies patients who have rare germline mutations that are often left untested in other community practice sites and may have a direct benefit to the patient if detected [46,47]. This can also help reduce the race-driven disparities seen in community practices where racial minorities are often not tested or given the option of genetic or germline testing during their cancer care [48,49,50]. Time constraints remain a challenge in both academic and network/community practices. In community practice, this has been shown to harm patient outcomes, often resulting from a hastened time taken towards treatment initiation without considering all informative diagnostic testing data, potential targeted therapeutics, practice gaps in evaluating the latest therapeutic research, and a lack of standardized research protocols including, but not limited to, clinical trials [51,52,53]. Developing and implementing the best treatment plan at the start of each therapy episode offers the best chance for improved survival and quality of life [54,55,56]. Our model directly addresses these limitations by providing very comprehensive pathways at all sites of enterprise care, promoting network physicians’ acquisition of knowledge and propensity to feel comfortable reaching out to individual academic specialists for informal and formal consultations, offering network clinicians participation with respect to their patients in disease-specific and precision oncology tumor boards, and providing regional COCDs. These COCDs allow the community practice leads to be at the forefront of selecting patient cases and requesting experts as needed without straining the geographic hub’s operations. To this end, the experts chosen to attend the complex case discussions are selected based on the individual cases that are challenging and require their expertise. Unlike traditional tumor boards where a large majority of cases are evaluated, our model allows the community practice oncologists to select only the complex cases. Our experience has shown that this heuristic approach to evidence-based learning can improve outcomes and overall network practice care [57]. Furthermore, our model helps address the time constraints of academic center oncologists that have experienced significant disruptions in consultations and care due to COVID-19 [58,59]. Our model limits the strain by lowering the number of consultations from network practice oncologists, which in turn provides value to the patient and lowers their costs without sacrificing care expertise. Network and academic oncologists who participate in complex case discussions are interested in obtaining academic and continuing medical education credits. The work towards such credit is under discussion. Both patients and network clinicians have reported a high degree of satisfaction with respect to knowing that the care plan for an individual patient has the best chance of offering the patient the best health outcome for their cancer diagnosis.

Value-based medicine is another key factor in our model that assesses not only the survival, toxicities, and financial costs to the patient from additional consultations and treatments but also takes into consideration personal values when establishing their plan of care. While costs of precision oncology continue to rise, with significant contributions from expensive genomic testing that ranges between USD 3000–6500 from commercially available sequencing platforms, the solution to this problem may be in implementing our approach in network practices where molecular data and genetic testing are performed based on granular evidence or clinical trials that are shared with the payer to justify the costs [60]. Such a model was slated to be adopted nationally through the Oncology Care First model and incorporated into CMS’s 2023 enhanced Oncology Medical Home (eOMH) model, where cost-savings may be dependent on the data presented to the payers for higher reimbursement [61]. Our model enhances the precision oncology promise in our network practices by allowing network physicians direct access and consultation to nationally and internationally recognized expertise without a requirement for a traditional consultation. This time- and cost-saving solution also allows patients to receive the latest available information and care, as many patients treated in the community do not obtain a second opinion and rely on their primary oncologist for the entirety of their cancer care [62].

The aim of this study is to detail and describe City of Hope’s pyramidal decision support framework for providing efficient and targeted support to busy clinicians in collaboration with expert faculty and to understand how any gaps in patient care can be further improved through academic and network practice collaboration.

## 2. Materials and Methods

A four-tiered pyramid of decision support was developed at the City of Hope to better serve a growing regional, national, and international network of cancer practices in this time of continuous rapid expansion of cancer diagnostics and therapy options. The 4 tiers are (1) evidence-based pathways via ClinPath, (2) formal and informal 1:1 faculty consultations, (3) 13 regular subspecialty or precision oncology tumor boards, and the newer (4) Complex Oncology Case Discussions (COCD).

**Evidence-Based Pathways (EBP):** Evidence-based pathways are a key component of City of Hope’s digital strategy for value-based care [5]. COH implemented the VIA—now Elsevier ClinPath—evidence-based pathways in January of 2017. Currently, ClinPath pathways provide standard-of-care treatment pathways for medical oncology, hematology, and radiation oncology for 29 diseases (22 solid tumors—breast, neuro, anal, colorectal, gastroesophageal, neuroendocrine, pancreatic adenocarcinoma, bladder, prostate, renal cell, testicular, ovarian, uterine, head and neck, thyroid, mesothelioma, non-small cell lung, small cell lung, melanoma, squamous and basal cell skin cancers, and sarcoma, and 7 hematologic—chronic myelogenous leukemia, immune thrombocytopenia, lymphomas, chronic leucocytic leukemia, myelodysplastic syndromes, multiple myeloma, and other plasma cell dyscrasias). The sarcoma pathways were added in April 2019 (Supplemental Figure S1). The pathways are determined by 19 disease committees of which COH faculty co-chair 4 (Breast, CNS, Gastroesophageal, and Bladder/Renal) and faculty with disease specialties participate in most committee meetings. Disease committees oversee navigations for common and, depending on committee consensus, add guidance or pathways for rarer tumors or germline mutations within pathways to provide deeper navigational guidance to clinicians. The initial goals were to provide guidance on common diseases with a goal of 80% pathway compliance. As complexities of molecular mutations and sequential therapies have evolved, there is a growing consensus regarding the addition of specific navigations for all evidence-based care to help busy clinicians, most of whom treat multiple types of cancer patients in a day.

At COH, the clinical informatics team has had a Pathway and Protocol Informatics Pharmacist (DJ) oversee monthly meetings with the academic disease leads and their specialty PharmDs, along with Epic Beacon builders and the value-based care medical director (LB). At these meetings, a standard agenda ensures a review of new FDA drug approvals or regimens, practice-changing therapies for customization and clinical trials that may need updated Epic Beacon regimens, and mapping from the ClinPath pathways to our Epic Beacon regimens.

A Pathways Committee was established before the initial go-live of VIA, now ClinPath, pathways in 2017. It has continued to meet monthly to review pathway use, on-pathway rates, off-pathway rates, and reasons for off-pathway choices by disease type and to review data reporting with Epic therapy orders. The group also oversees the recruitment of members to serve on-pathway disease committees and oversees improvements in Epic–ClinPath interfaces and clinical trial integrations.

COH clinicians or their team members document pathway navigation choices in one of two ways. A total of 30% of clinicians navigate to the pathway tool through our electronic health record when they plan to order a systemic medical oncology or hematology therapy (other than BMT, cellular therapies, or acute leukemias). By using an “order with pathways” link, the staging and biomarker data from the EPIC-staging forms are populated into the pathway tool. The clinician then only adds any additionally required information before being taken to the preferred treatment options, which start with our clinical trials. If a pathway choice is made, the clinician is then taken back to the mapped Epic Beacon treatment orders, which include the NCCN-compliant antiemetics and any other disease lead team-determined guidance. The second option for clinician navigation is to order their preferred therapy in Epic Beacon, and directly after which we ask that, asynchronously, they or their staff enter the regimen’s pathway navigation information into the ClinPath tool. Elsevier provides a monthly report of pathway navigations by site, doctor, and disease, reporting choices for On-pathway, On-pathway-off treatment, Clinical Trial, and Other Trial, of which all 4 are considered On-pathway. Reporting also includes the other navigation option: Off-Pathway. Direct data feeds from ClinPath to our EDW populate Tableau reports. These reports show the 5 choices as well as when a provider enters the Not a Pathway diagnosis, No Pathway, and Off treatment choices. Clinicians are prompted to enter a reason for Off-Pathway choices. Tableau dashboard reports are sent to clinicians weekly to show any missing navigations to encourage completion. Other reports are sent to leadership showing pathway compliance by disease, site, region, and physician. Institutional or departmental incentive programs have encouraged at least 80% of ordered therapies for covered diseases to be navigated in the pathway tool. Payer metrics are incentivizing the enterprise to more fully capture available pathway navigations as well.

**Formal and Informal Faculty–Network Clinician Consultations (FCC):** Communication among faculty members is essential for supporting busy oncologists who see multiple types of cancers each day to optimize patient care. An opportunity for the growing networks of academic and network oncology clinicians is the establishment of true respect and collegiality. This has been a key goal of COH’s enterprise and chair leadership. In medical oncology, regular symposia where academic and network clinicians co-present on cutting-edge topics have brought collegiality, respect, and awareness of specialty expertise and clinical challenges among the faculty. The option of formal consultation that can be provided to services both from academic faculty to colleagues on the academic campus and by network clinicians to academic campus clinicians when deemed necessary for any patient’s care is available to all faculty. Additionally, network clinicians feel very comfortable reaching out to expert faculty on clinical issues when an informal 1:1 consultation can resolve a question. Academic faculty have also become comfortable sending their patients back to network sites for care to minimize travel time and facilitate more involvement of family and caregivers in local communities. While we can track formal consultations from medical oncologists to academic site faculty and academic faculty consultations to other academic colleagues, we currently have no formal method to collect the number of informal consultations that occur, the specific faculty, clinical issues raised, nor the impact on changes in the care plans.

**Tumor Boards (TB):** Over the three years studied, for selected cases where academic and network oncology clinicians desired additional decision support beyond EBP, COH has offered 13 weekly or biweekly multispecialty, disease-focused (11), molecular oncology/precision oncology (1), and multi-tumor (1) boards (TB). Traditionally, tumor boards were focused on specific organs, e.g., breast cancer. However, cross-cutting tumor boards, such as molecular tumor boards, are important for patient care and research [63].

The composition of disease-specific tumor boards includes the traditional disciplines of providers including a surgeon, medical oncologist, and radiation oncologist, as well as pathologist and radiologist, while the Precision Oncology tumor board has a higher number of geneticists and non-physicians with expertise in detection and discovery of the molecular composition of cancer. Total attendance at each TB meeting is recorded, though data on the subspecialties of all attendees were not specifically recorded.

Cases are submitted in advance to the tumor board. Submissions include relevant case information and any specific questions the submitting provider has for the multidisciplinary team. Cases are reviewed ahead of time by pathology and radiology specialists and additional materials such as digitized pathology slides are prepped for presentation. Similarly, images are loaded for review and discussion during the meeting. Tumor boards are also recognized as a forum for identifying the most appropriate place for patients to receive any elements of their care that require a highly specialized setting in coordination with care delivered at a network site selected by the patient or their health plan. For example, highly specialized surgeries are directed to the academic campus as are potential candidates for clinical trials when they are not open at a closer network site.

Until March 2020, TBs were held in person, which limited the ability of network oncology clinicians to attend. Subsequently, due to the COVID-19 pandemic, TBs were made virtual, leading to potentially improved accessibility [64]. Although TBs have been able to provide a critical additional level of decision support, we currently do not have a formal procedure across all TBs for reporting on submissions, the context of discussions, and specific recommendations. Nor do we have information as to whether the proposed care plan was approved, adopted, or revised [65], which limits the quantification of the overall impact of these TBs on patient management. Efforts to improve this deficit have come from this study and are the focus of a new institutional quality improvement project, which will help ensure TBs are both efficient and have reportable impacts on patient outcomes [11].

Our tumor boards, like most, vary in their content, disease focus, and membership composition [66]. Beyond the time commitment for faculty attendance, resources are committed to coordinating and preparing for the meetings. To understand the structure and process of these tumor boards, we collected data on the schedule of the tumor boards, membership and attendance, operating procedures for coordination of the tumor boards, data management for tumor boards, and potential patient impacts. Additionally, we used the alteration in the operating procedures during COVID-19 as an opportunity to assess any changes in the attendance at tumor boards and the opportunity for modernizing the concept of tumor boards [67]. Available case data from each tumor board that occurred from January 2019 through December 2021 were collected from respective TB administrators. TB attendance was collected from the Continuing Medical Education department.

**Figure 2:**
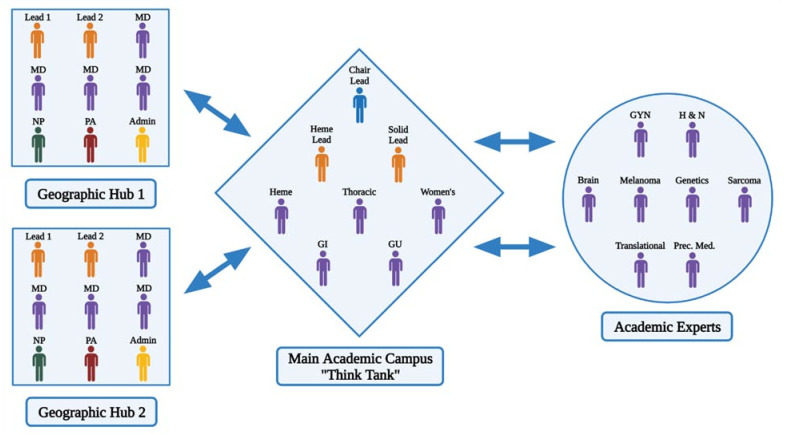
Complex Oncology Case Discussion: community practice hub and academic site ‘think tank’ integration of expertise.

**Complex Oncology Case Discussions (COCD):** The experience of our oncology practices during the COVID-19 pandemic highlighted a gap in our decision support offerings with a need to streamline network practice consultations due to the limitations of in-person referrals, the geographic growth of network sites, and growing care complexities, as noted by others [11,68]. We thus developed an additional level of faculty decision support to network clinicians called Complex Oncology Case Discussions (COCD). These are led by regional network practice sites in collaboration with the academic center. COCDs are constitute a multi-faceted approach to sharing academic site expertise in a timely and practical fashion with network physicians (Figure 2).

The challenge in creating and establishing this model was in leveraging the availability of very busy individual team leaders and subspecialty experts with the very busy network practice physicians for regular weekly, bi-weekly, or monthly COCD. To overcome this challenge, our model was established so that the site leads of the individual sites assess their cases, determine the expertise that is required before the meeting, and only include individuals for the regional complex oncology case discussion as necessary based on their expertise. Every network practice site is assigned two site leads who supervise and organize the needed faculty experts for regional discussions with the network physicians. Our original academic site in Duarte includes a thinktank of disease team experts from oncology and hematology with access to chair leads of the various disease teams. This collaborative conference between the network and the academic site allows the oncologists to receive expertise from sub-specialized oncology experts, which would otherwise be absent and can be missing in pathways for common disease presentations as well as traditional tumor board models [69]. Evidence has shown that integration of such a collaborative model increases survival and outcomes in community practice and allows for expert intervention in situations where the network practice cannot provide the care or expertise required based on individual complexity [70]. Therefore, our model enhances the paradigm of precision medicine through the inclusion of individual disease team experts including rare diseases such as sarcoma and head and neck cancer, as well as the implementation of the expertise of a genomics expert and genetic counseling in the network practices.

In practice, this has been implemented efficiently and with minimal disruption of both the academic site and the community practice physician’s schedules. We now have 3 regional COCDs that meet monthly and as needed whenever urgent COCDs are needed. We believe that the fluidity and structure of our multi-faceted team of experts have the potential to transform network practice interactions and traditional consultations into necessary functions for all our network site practices to provide as-needed, seamless, state-of-the-art academic inputs for precision care plans for patients. By only gathering the needed experts for each COCD, we optimize the demands on faculty time and have focused meetings to meet the specific needs identified by the network clinicians [71].

**Figure 3:**
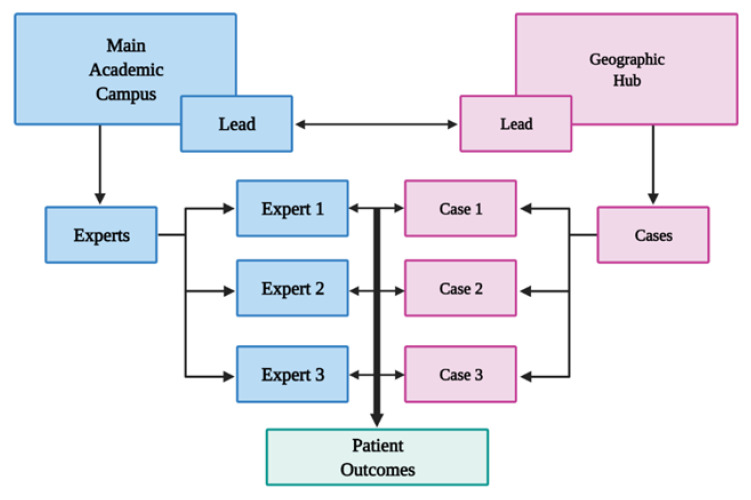
Complex Oncology Case Discussion algorithm for expert collaboration between academic and geographic network hubs.

Process-wise, two leads at the regional network site along with the site administrator gather the individual cases and any specific questions from the regional network practitioners before convening the COCD. The leads assess the individual cases and determine the expertise that is required to attend the COCD and notify these experts 2–5 days before the meeting. The network practice physicians and administrators work with the academic site thinktank supervised by the disease team leaders and the chairs of the departments to invite the experts requested. While 4–6 leads are permanently assigned to the multidisciplinary team, other leads and experts of rare diseases such as brain cancer, sarcoma, melanoma, and head and neck cancers are invited as needed. The COCD is then convened virtually, and cases are assessed to answer any questions and determine the plan of care based on the consensus between the network practice and the academic site physicians (Figure 3).

If a plan of care cannot be decided due to a lack of further information such as a pending or recommended genomic-testing procedure or additional biopsy, then the network practice physicians can follow up with the academic site experts on the individual cases. Further consultation and intervention are available both virtually and in person for cases that require genetic counseling, radiation oncology intervention, specialty surgery, and clinical trial consultation. The integration of network practice sites into the academic site model also allows for clinical trial screening and trial onboarding at a few larger designated network sites—with the option for the patient to receive the trial drug treatment at the network site or the academic site. If the patient eventually relapses or undergoes progression, the network site leaders can alert the thinktank experts to convene another COCD or refer them directly for an academic consultation. This model allows for the fluidity of care and provides the patient with a consistent primary oncologist while maintaining state-of-art care that is associated with academic centers. We hope that the implementation of this model will result in greater survival outcomes as compared nationally and as have been seen at our academic site with the potential to transform networked practice care nationwide [23,24,25,26,27].

## 3. Results

The results of COH’s Pyramidal Decision Support Framework will be shown for each component. The overall survival analytics have shown superior survival data for analytic patients for all stages and cancer types seen at City of Hope’s Duarte academic campus compared to regional and national SEER data and published on our site for breast cancer, lung cancer, colorectal cancer, prostate cancer, and myeloma [23,24,25,26,27]. City of Hope began adding regional cancer care network sites in 2010, which grew to 5 sites in 2011, 17 by 2014, and 30 by the end of 2018. During the 3-year period, 2019–2021, City of Hope expanded from 30 to 38 network sites, which are included in this analysis. These sites were served by 29 academics, Duarte campus medical oncology faculty, and 50 network medical oncology faculty. The network medical oncologists also saw hematology patients. These clinicians saw 8479 new, 3752 consults, and 38,263 unique patients when 25,429 follow-up visits were included in 2019. They saw 6637 new, 2836 consults, and 43,329 unique patients when both 10,711 telehealth and 22,722 follow-up visits were counted in 2020. For 2021, the group saw 8807 new, 2915 consults, and 56,143 unique patients when 13,229 telehealth and 30,775 follow-up visits were counted.

A value framework has been built to track patient information, therapies, pathway choices, and survival for analytic and non-analytic patients seen across the enterprise since the implementation of our EPIC system in December 2017. Data are being tracked for 5- and 10-year survival outcomes but are not yet mature. This framework will provide clinical outcomes for patients whose treatments were guided by these four components of decision support. We present the initial use data on the most recent 3-year period: January 2019 through December 2021.

**Evidence-Based Pathways (EBP):** The data sent from ClinPath to our enterprise data warehouse (EDW) weekly are presented in Tableau reports according to the clinician, practice, region, network, and academic site, and according to the navigation choice for enterprise, Duarte academic center, and the network as well as its individual sites. Disease navigations can be reported for quarterly, annual, and time-bounded periods.

We have built Tableau charts so that clinicians and administrators can review all decisions by pathway disease. The On-pathway rates can vary significantly by disease and time period. The original goal of the pathway system was to cover 80% of therapy choices. Given the rising importance of understanding why a therapy was prescribed for a specific patient and their disease, some pathway committees have expanded guidance and flow sheet options to cover more episodes of care to provide a national group of cancer experts recommendations for the best options when appropriate. Not all committees have adoptedthis approach, so the on-pathway compliance rates will vary by how fast a national group adopts practice-changing information from presentations to publications and on to FDA approval and health plan coverage as well as by the depth of evidence-based recommendations the committee feels warranted. At City of Hope, we have no prespecified on-pathway compliance expectations but want the pathway system to provide the best standard care recommendations for the increasingly more complex range of therapy choices, especially as patients with some cancers benefit from more than three to four lines of therapy and since the therapies they had previously in any setting impact the currently recommended best option along with evolving molecular genomic and other diagnostic tests.

The evaluation of pathway navigations for the 22 solid tumors with pathway choice data from our Tableau system for the 3 years, January 2019 through December 2021, reveals over 35,000 pathway decisions across all seven decision categories: No Pathway/Not a pathway diagnosis, On-Pathway, On-Pathway–Off treatment, Clinical Trial, Other Trial, Off-Pathway, and Off Treatment (data available but not shown). Almost 15,000 were performed in network sites and over 20,000 were performed at Duarte’s academic site with up to 28% of network and 8% of academic sites noting a non-pathway diagnosis. Of these total decision types, the overall on-pathway choices corresponded to 35–45% at Duarte and 38–60% in the network sites when there was a disease pathway, which is due to the percentage of other-than-On/Off-Pathway choices. Some diseases were noted to have low on-pathway rates for some quarters, which suddenly changed in a subsequent quarter. Investigating these changes showed the pathways had been updated through regularly set meetings that included key updates that our academic leads had already implemented. What had been considered ‘off pathway’ can change to ‘on pathway’. This time delay from the early adoption of practice-changing reports and research can influence the timing of what is considered off- vs on-pathway and remains a challenge to harmonize.

**Figure 4:**
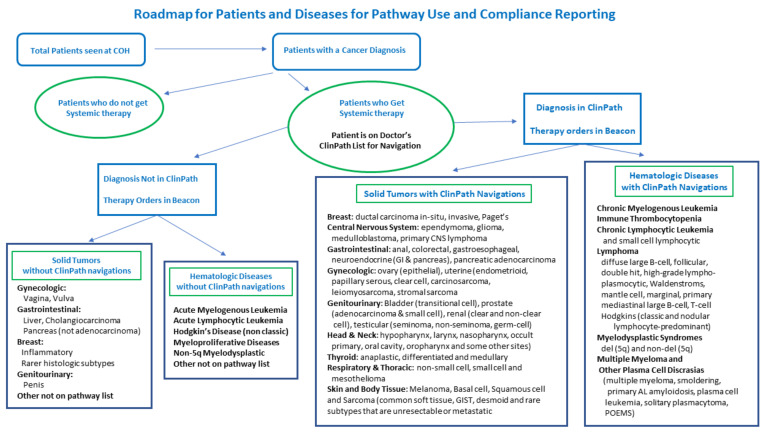
Roadmap of patients seen at City of Hope to understand those with diseases eligible for pathway use and reporting from ClinPath system. Both patients with and without a diagnosis in the ClinPath system will have Beacon therapy orders in the EHR. GI (gastrointestinal), CNS (central nervous system), GIST (gastro intestinal stromal tumor).

The data from ClinPath on navigation choices for patients with diagnoses that have a pathway in the system are the largest subset of patients who receive systemic therapies at City of Hope. Figure 4 shows how patient data flows from all patients to those who get systemic therapies and from that subset, which diseases and their subtypes have a pathway in ClinPath for navigation and which do not. For diseases without a pathway, such as cervical cancer, hepatobiliary disease, myeloproliferative disease, ALL, AML, cellular therapies, and other rare, advanced line or very rare mutation-related diseases, a no pathway available category is available to enter in the pathway tool; however, most doctors who treat those diseases order therapies for non-pathway diseases and subtypes directly in the EPIC Beacon EHR and they may not be available from the navigation data. We are in the process of building new databases in our EDW to study the disease, stage, biomarker, line, type, ECOG, and therapy ordered for every patient seen. We can then divide those into diseases and settings with standard pathways available to pair with pathway choice navigations and track those without pathway tool pathways available. The addition of sarcoma pathways in 2019 was highly advocated and supported by City of Hope faculty given the numbers we see and the expertise we felt would be beneficial to have in our formal pathway system.

**Figure 5:**
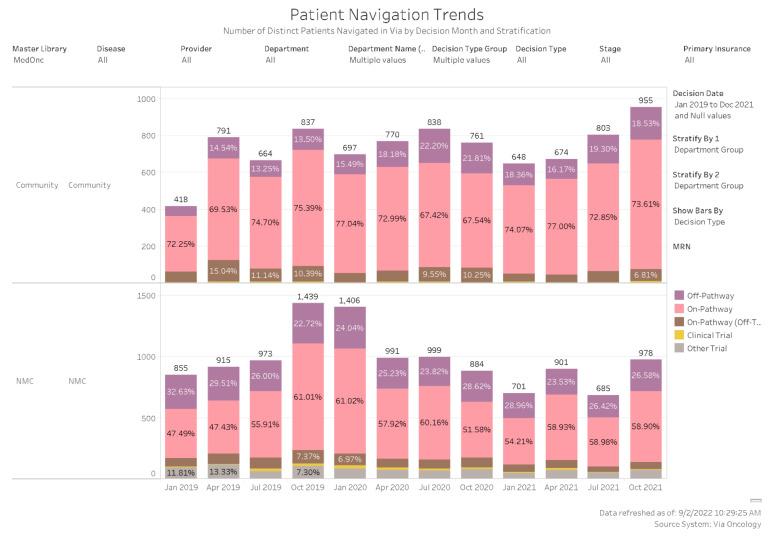
ClinPath Tableau Report of On-pathway vs Off-Pathway choices for covered solid tumors and hematology diagnoses by quarter in Duarte and Network sites for 1 January 2019–31 December 2021: Off-Pathway and On-pathway, (On-pathway, On-Pathway-Off Therapy, Clinical Trial, and Other Trials) navigation choices.

Reviews of the more commonly reported Off-Pathway vs. On-pathway (On-pathway, On-pathway-off treatment, Clinical Trial, and Other Trial) choices for patients who were navigated through ClinPath for the 29 covered diseases (22 solid tumors and 7 hematologic) from our Tableau system for the 3 years (January 2019 through December of 2021) are shown by quarter in Figure 5 for the academic center and network sites. These results are summarized in Table 1 for On-pathway vs. Off-Pathway results from the enterprise, academic, and network sites. The data show that there were 20,583 total On/Off-Pathway Decisions for the enterprise over the 3 years, of which 79% were On-pathway. A total of 8856 decisions were made at Network sites, of which 7324 were On-pathway for an 83% rate, while 11,727 total decisions were made in Duarte with 8901 being On-pathway for a 76% on-pathway rate.

Data by quarter can also be presented for the 19 solid tumor types in the 10 categories (breast, GU, GI, GUN, Head and Neck, Skin, Neuro, Neuro-endocrine, Lung, and Other) and for the eight commonly seen hematologic diseases with pathways in ClinPath (CML, CLL/Lymphoma (B Cell, T Cell, and common histology Hodgkin’s), multiple myeloma, MDS, and ITP). Table 2 shows the on-pathway rate, the total number of on-pathway decisions, and the total decisions for 18 of the most common pathway diseases, where we had at least 200 decisions or an on-pathway rate of ≥80%. Upon review, breast, pancreatic, gastroesophageal, melanoma, neuro, bladder, and testis cancers all met the goal of ≥80% on-pathway therapies. Renal and colorectal cancers were on-pathway 78% of the time. An internal review of these, as well as the non-small cell’s 64% on-pathway rate and small cell’s 67% rate, reflect the rapid changes in therapy recommendations during these 3 years such that at the time of decision making, COH’s disease lead choices were ordered, which was only later reflected in the pathway updates. There is no current report to compare off-pathway decisions that would, in a subsequent quarter or time period, be considered on-pathway. Such a report could show the early adoption of practice-changing therapies before their incorporation into pathway tools. This remains a challenge for the reporting of pathway data. Of note, each of the most common hematology diagnoses, multiple myeloma, and CLL/lymphomas had over 80% on-pathway choices made for their therapies, for which the reports are almost solely from network sites, as our academic colleagues plan to expand their use of the hematology pathway tool in the future.

We also collect data on navigations for the eight commonly seen hematologic diseases through the pathways in ClinPath (chronic myelogenous leukemia (CML), chronic lymphocytic leukemia (CLL)/Lymphomas (B Cell, T Cell, and common histology Hodgkin’s), multiple myeloma, myelodysplastic syndromes (MDS), and idiopathic thrombocytopenic purpura (ITP) and can generate Tableau reports. Network clinicians have valued and navigate care through the hematology pathways while academic center clinicians have only recently started using hematology pathways. The 3-year data, from 1 January 2019 to 31 December 2021, for network physicians’ use of the pathways shows that 968 patients from network sites had therapies ordered for hematologic diseases with a range of 70–95% of decisions were On-pathway choices. Of note, ITP, a benign disease, is included in our pathways, as there are multiple very costly but effective options for therapies for this commonly seen disease in our network sites. Starting in a cost-effective sequence ensures that patients obtain the therapy with the best chance at efficacy and with the lowest cost or toxicity. Severely refractory patients often receive a long sequence of therapies, so choosing the most cost-effective approach at each step can lower their overall cost and improve quality of life by using oral therapies and those requiring fewer clinic visits early on and hoping that most will not need every type of therapy available.

As we engaged our academic disease leads for hematology and medical oncology, they identified many regimens that had not been built in our Epic Beacon system. Of approximately 900 regimens in our Epic Beacon system, 638 are regimens in the pathway system. Working with our disease leads in 2021 and 2022, we identified 300 therapy regimens that needed to be built, modified, merged, or mapped from the pathway tool into Epic to fully integrate our pathway and ordering system. 220 have been completed with 50 more due to be completed in December 2022 and the remaining 30 by February 2023. Completing standardized regimen builds in the EHR provides clinicians with a robust and efficient pathway ordering process from the pathway decision prompt. Standardized therapy orders support include the therapy agents, dosing and schedule as well as partnered antiemetic regimen by emetogenic risk level, laboratory, nursing and education visit orders to efficiently facilitate pre-authorizations, patient education, care delivery, payment metric reports and internal analytics. Two updates in progress will have the ClinPath team placing new clinical trials weekly into our pathways while an OnCore integration will provide real time status updates for clinical trials in the pathway tool. Clinical trials appear first in the pathway options and will be shown as pending, open, on hold or closed. These upgrades are expected to improve trial considerations and accruals.

**Faculty Clinician Consultations**: Formal consultations between the academic medical oncology clinicians to other academic faculty across disciplines totaled 4083 in 2019, decreased to 3978 consults during the first pandemic year (2020) and rebounded to 5635 consults in 2021 as the pandemic was mitigated in our region. The main specialties consulted over the 3 years from the academic medical oncology faculty were the surgical oncology specialties with 6119 consultations followed by radiation oncology with 3401 consultations and hematology with 703 consultations.

From the network of medical oncologists, there were 6203 consultations in 2019 referred to campus specialists in medical oncology (4883, 81%), surgical oncology specialties (744, 12%), hematology (393, 6%), and radiation oncology (91, 1%). By 2020, there were 5623 consultations in similar ratios for these main oncology specialties. This reflects that most network sites have City of Hope surgical and radiation oncology specialists locally who provide specialty oncology care. Thus, most consultations provided by medical oncologists to the academic center are for medical oncology to collaborate on complex patient presentations or clinical trials not offered in the community. Of note, there was only a 10% drop in overall consultations in 2020, which included the time after the global and regional COVID-19 pandemic was announced in March of 2020. This shows that despite the pandemic, cancer services could still be provided with the comprehensive safety measures instituted at the campus and network sites to protect staff, patients, and their families. By 2021, the third year of our study, similar tableau reporting on consultations from network medical oncologists provide to campus faculty showed a significant drop to 1494 consults overall, which is a 76% drop from 2019 and a 73% drop from 2020. These major decreases occurred in medical oncology consultations for gastrointestinal, breast, genitourinary, gynecologic, and thoracic subspecialties. The exact reasons for this drop have not been studied but are postulated to be from the expanded access to 1:1 informal faculty consultations, tumor boards, and complex oncology case discussions that that is providing high-quality input between the academic and network medical oncologists to meet patients’ care-planning needs.

Data were not collected for the many informal consultations that occur between network and faculty clinicians nor for the questions raised, any changes in workup or care plans recommended, nor the potential impact on those discussions. Even though formal consultations result in consultation notes and communications, we do not currently track the impacts of recommendations from those consults. Most of the 29-member academic medical oncology faculty report at least one call per week from network clinicians and colleagues regarding oncology care planning for an oncology patient. Over 52 weeks, this would represent 1508 informal consultations or over 4500 consultations over 3 years. Our study identified this as an opportunity for improvement and to define and collect data on numbers, issues, and likely impacts, as well as the time invested for both formal and informal consults, in order to better understand the academic specialty faculty workloads and return on invested time.

**Tumor Boards:** A review of the available TB data demonstrated significant variability in the discrete data points collected and how the data were stored. A total of 4653 cases were presented across the eleven TBs. All cases contained the submitting provider’s name and at least a general case description and question for the TB, though there was significant variation in the structure and extent of these data points. Case recommendations were recorded in six of the TBs, corresponding to 63% of all cases. However, whether these recommendations constituted a change from the initially proposed course of management was not recorded. The composite TB data that could be queried were only available for the Musculoskeletal Sarcoma TB, representing 8% of the total cases. This TB was led by only one disease expert since its inception, who established the methodology and oversaw the data collection. The data for other TBs were stored in the form of text documents or PDFs, which limited quantitative analysis (Table 3).

**Figure 6:**
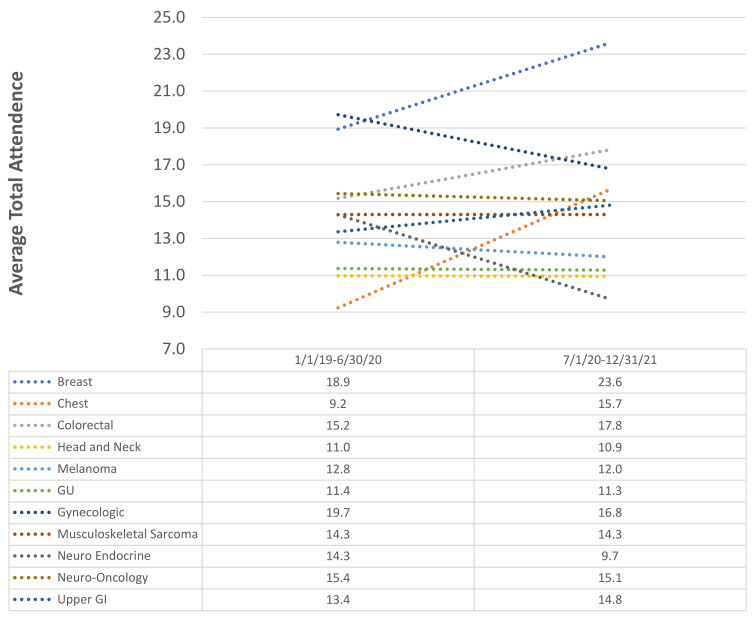
Tumor Board Attendance Over 3 years: 1 January 2019 to 31 December 2021.

To characterize the impact of the transition from in-person only TBs to virtual TBs, the average TB attendance was compared between the 1 January 2019–30 June 2020 time period and the 1 July 2020–31 December 2021 time period (Figure 6)**.** Absolute changes in the attendance of between −1 to 1 participant were considered stable. Of the eleven TBs, the attendance at four TBs increased while it remained stable in five TBs and decreased in two TBs. The largest increase in attendance occurred in the breast TB, with an average increase of 4.7 attendees (*p* < 0.001), while the largest decrease was −4.6 attendees in the neuroendocrine TB (*p* < 0.001). On average, across the eleven TBs, attendance increased by 0.6 attendees (*p* < 0.001).

There is no over-arching standard for the data collection or reporting of TB presentations nor a mechanism for tracking adherence to tumor board recommendations. The presenting faculty takes responsibility for executing the plan discussed in the tumor board. Another challenging issue is if more information is needed to render a final plan of care proposal, the re-presentation of the patient is not universally pursued, which could limit the potential for additional multidisciplinary decision making for this subset of patients.

**Complex Oncology Case Discussions**: We currently have three different Complex Oncology Case Discussions across the network of City of Hope. These meetings are organized regionally and have been held monthly with an option for urgent COCDs if needed. Table 4 shows the COCD data. COCDs’ started at the Newport site, which now involves two other regions of practices that joined the network in 2020. As word spread on the value of COCDs to clinicians and patients, a second region started regular meetings in August 2020 and a third region started in March 2021. All are now held regularly with 14–21 attendees of which 4–10 are from the academic faculty. When answering questions for this study, the COCD leads uniformly described the COCDs as of high value to target complex questions very efficiently and completely. They also reported high patient satisfaction and peace of mind knowing their complex cancer diagnosis had been reviewed by specific experts to determine either the need for any additional workup, the option for a clinical trial, or the development or confirmation of a personalized cancer care plan. The Newport group highly values the involvement of their radiation oncologist as well as their onsite radiologist in the reviewing of films as needed. Other regional COCD directors hope to add such expertise as needed over time. COCDs’ attendance is less diverse than that of tumor boards. These meetings are not supported by the pathology services, partly due to access to source materials. All patients receiving care at City of Hope, however, are required to have their pathology reviewed by COH pathologists. The case discussions are primarily focused on medical oncology interventions and transitions of care across lines of treatment as well as candidacy for clinical trials. The meetings are currently coordinated by the network physicians without using administrative staff. The format enables in-depth discussions, the engagement of the providers in the network practices, and the optimization of care via knowledge transfer. With these new meetings, we identified that having a standard intake-reporting form and meeting summary report with standardized categories of discussion and recommendations with possible likely impacts would be of value to the regional leads and the COH leadership. Discussions of a standardized format for such reporting are underway.

## 4. Discussion

City of Hope’s enterprise commitment to democratizing precision cancer care includes the provision of multispecialty, cutting-edge cancer knowledge to the chairside of every network clinician. With the rapid expansion of the City of Hope network regionally and now nationally, this goal is being realized through our pyramid of decision support. This pyramid expands on traditional evidence-based pathways, formal and informal faculty consultations, and tumor boards via its added Complex Oncology Case Discussions that bring rapidly evolving knowledge to clinicians so they can enhance the provision of customized cancer care plans to patients whose cancer diagnoses do not have standardized therapy approaches. The study of this framework—which has evolved over the last 4 years as we continue to expand our care delivery network regionally, nationally, and internationally—has identified strengths and opportunities to fill further gaps in understanding the efficacy and impact of these tools.

The pyramidal decision support project falls within our evidence-based care pillar in our comprehensive value-based care framework. These initiatives have been discussed previously [5]. They are supported by a comprehensive digital data strategy so as to have all discrete data within our enterprise data warehouse so that informative analytics can be made available to meet our expanding geography of care-delivery sites, expand access to clinical trials and state-of-the-art cancer care, achieve identified quality-of-care goals, and support our growing oncology-focused medical-home-type payor contracts.

The pathway improvements have come from the establishment of a formal program to capture disease leads directing new drugs and therapy builds in our Epic EHR, overseeing the addition of clinical trials, validating ClinPath recommendations, and identifying any customization to achieve the best outcomes for patients. New operational initiatives to incentivize real-time pathway navigation when ordering systemic medical oncology and hematologic therapies have been identified, as is performed by our radiation oncologists. This will improve the capturing of pathway choices for each line of therapy ordered, which can improve prior-authorization turnaround times, enhance analytics to support growing medical-home-type payor contracts, and inform our quality reporting and the disease leads of therapies being given throughout the enterprise for specific cancer subtypes. The improved discrete data capture of all entered elements from the pathway decision tool to our enterprise data warehouse (EDW) is underway to improve the validation of Epic and ClinPath data, which will support expanded value-based analytics.

Network and academic clinicians have long had the option to reach out to colleagues with subspecialized clinical and research expertise for the 1:1 discussion of patient issues and to order formal consultations when the standard of care is not applicable or optimal. We do not currently collect any data on these informal but very helpful consultations. However, we have data on formal requests for consultation. The consideration of a simple report of informal consultations with clinicians, patient issues, recommendation categories, and likely outcome impact via an efficient EHR tool or phone app might further capture valuable work performed by subspecialty cancer faculty for which they currently do not receive recognition, time, or compensation.

Disease-specific and precision oncology tumor boards carry out essential work by bringing multidisciplinary teams together to ensure that the care plans of presented patients are optimized. Given the substantial resources required to provide these tumor boards, we identified a need to understand the full impacts of this resource more formally. Our study led to the development and launch of a quality improvement study to capture (1) structured data about the issues raised for the patients presented, (2) attendees noting academic and network clinicians by specialty, and (3) the capture of structured decision impacts. Enhancing standardized data collection can better inform the enterprise about the impact of the many clinician and staff hours invested to improve patient care planning for patients’ best health outcomes. The questionnaire for the study is shown in Supplemental Table S1.

Our novel Community Oncology Complex Discussions have been a welcome addition to our pyramid of decision support offerings. These discussions meet the requirement for providing the urgent expertise of academic site specialists who can be flexibly distributed to network practices without interrupting either the academic or network sites’ operations. This integration is becoming more important as our enterprise grows to serve more diverse and geographically distant practices where general oncologists and their patients welcome input from subspecialty faculty who can rapidly share the newest therapeutic options, provide expert evaluation and recommendations for germline mutational-testing and results, advise on a clinical trial of a new agent, an agent available for compassionate use, or one not yet approved for an expanded indication. The impact of these complex decision-making discussions may provide patients with new therapeutic options from targeted therapies, to immune, or cellular therapies that may not be available or known to network clinicians but may significantly improve patients’ cancer outcomes.

Our COCD model, however, could be further enhanced through cross-institutional collaborations for orphan diseases and rare tumor sub-types such as NUT carcinoma (‘nuclear protein in testis’ carcinomas, which can be found anywhere in the body but are often midline), which require national and international expertise to arrive at proper clinical trial options for this vulnerable population of patients. At the same time, there is potential to extend this model beyond clinical operations and integrate network practice physicians and patient data and specimens into research operations where specimens can be collected into a network-wide tumor bank that evaluates patients for potential new clinical trials based on the molecular and research-derived testing results [72]. Our institution has currently made strides in implementing this strategy of an institution-wide tumor bank for tissue samples and genetic results, including germline-testing results, but further efforts are required to ensure that all patients are captured in this model. The enhancement of the decision support pyramid with the COCD component has the potential to enhance and transform the enterprise practice of cancer care across the nation and allow for seamless, transformative precision medicine care for patients without the need for the traditional consultation model.

## 5. Conclusions

Decision support that can be efficiently and effectively provided in real-time or near real-time to every clinician before finalizing a patient’s care plan for each episode of their cancer care has the best chance of optimizing patient outcomes across a spectrum of measures. Comprehensive decision support with integrated tools to share current and cutting-edge knowledge can improve diagnostic testing, identify the most effective therapies, and reduce toxicities and avoidable emergency room and hospital admissions, which can improve patient and providers’ satisfaction and goal-concordant-end-of-life care. As payers move to more accountable, metric-based incentive contracts, having tools that incorporate reportable metrics and bring subspecialty faculty expertise to every network clinician can be informed by our Pyramidal Decision Support framework, which filled an unmet need with the addition of the COCD component. As enterprises such as City of Hope grow to expand access to high-quality cancer care, pyramidal decision support tools serve as critical components to democratize cancer care efficiently and with measurable outcomes.

## Figures and Tables

**Table 1:** Therapy Decisions from ClinPath Tool for Solid Tumors and Hematology by Enterprise, Duarte, and Network Sites. Pathway Compliance for Enterprise (E), Duarte Academic Campus (D), and Network (N) Clinician ClinPath Navigations over 3 years, 2019–2021. Total Number of Decisions On and Off-Pathway; total number of On-pathway Decisions for Enterprise, Duarte, and Network Sites; and total percent of On-pathway Navigations for Enterprise, Duarte, and Network sites. From ClinPath Reports.

Total # On/Off-Pathway Decisions			# On-Pathway			% On-Pathway		
E	D	N	E	D	N	E	D	N
20,583	11,727	8856	16,229	8901	7324	79%	76%	83%

**Table 2:** Enterprise 3-Year ClinPath Navigation Data: Percent (%) of On-pathway Decisions, total number (#) on-pathway decisions, and total number (#) of decisions by 18 tumor types with >200 decisions or >80% On-pathway rates. Tumors are ranked by the number of decisions from the highest number for breast cancers down to the lowest for testicular tumors. A blue highlight indicates On-pathway rates > 80%. Tumor type CLL is chronic lymphocytic leukemia, Neuro refers to brain tumors.

3-Year Enterprise On-Pathway Data			
Jan 2019 through Dec 2021			
Tumor Type	% On Path	# On Path Decisions	Total # Decisions
Breast	85%	5946	6954
Colorectal	77%	1834	2384
Non-Small Cell Lung	64%	1486	2317
Pancreatic	91%	1026	1124
Ovarian	70%	653	937
Gastroesophageal	81%	678	838
Prostate	91%	738	814
Head and Neck	69%	418	603
Lymphoma and CLL	83%	491	593
Uterine	67%	361	538
Melanoma + Skin	74%	366	494
Neuro	96%	355	369
Multiple Myeloma	88%	308	351
Renal	75%	242	325
Sarcoma	74%	226	304
Bladder	84%	251	300
Small Cell Lung	72%	153	214
Testicular	87%	77	89

**Table 3:** Tumor board data collected for 11 Multidisciplinary Tumor Boards Over the 3 years: 1 January 2019 to 31 December 2021.

Conference	Total Cases	Submitting Provider	Case Description	Specific Question	Case Recommendations	Data Centralized/Readily Accessible
Breast	585	Yes	Yes	Yes	Yes	No
Chest	600	Yes	Yes	Yes	No	No
Colorectal	326	Yes	Yes	Yes	No	No
Genitourinary	303	Yes	Yes	Yes	Yes	No
Gynecologic	521	Yes	Yes	Yes	Yes	No
Head and Neck	786	Yes	Yes	Yes	Yes	No
Melanoma	262	Yes	Yes	Yes	No	No
Musculoskeletal Sarcoma	375	Yes	Yes	Yes	Yes	Yes
Neuro-Endocrine	199	Yes	Yes	Yes	No	No
Neuro-Oncology	324	Yes	Yes	Yes	No	No
Upper Gastrointestinal	372	Yes	Yes	Yes	Yes	No
Total (%)	4653 (100%)	4653 (100%)	4653 (100%)	4653 (100%)	2942 (63%)	375 (8%)

**Table 4:** Details of comprehensive case discussion conferences.

	Orange County	Inland Empire	North Valley
Start	June 2020	August 2020	March 2021
Frequency	Monthly	Monthly	Monthly
Format	Virtual	Virtual	Virtual
Community Sites	Newport Beach, South Bay, Irvine	Upland, Corona, Arrowhead	Antelope Valley, Santa Clarita, Mission Hills, Thousand Oaks, Simi Valley
Attendees	14 (4 Duarte campus)	21 (10 Duarte campus)	16–20 (6–8 Duarte campus)
Cases Presented Per Meeting	4.5	4	5

## Data Availability

Not applicable.

## Supplementary Materials

The following supporting information can be downloaded at: https://www.mdpi.com/article/10.3390/jcm11226738/s1, Figure S1: Tumor Types by Disease Categories in City of Hope’s ClinPath Pathways. Table S1: Tumor Board Quality Improvement Questionnaire.
