# Supplementary material for: Pyramidal Decision Support Framework Leverages Subspecialty Expertise across Enterprise to Achieve Superior Cancer Outcomes and Personalized, Precision Care Plans
Source: J Clin Med. 2022 Nov 14;11(22):6738. doi: 10.3390/jcm11226738 (PMC9697355; doi:10.3390/jcm11226738)
Supplement: Supplementary file 1 [file jcm-11-06738-s001.zip › jcm-1971934-supplementary.pdf]

Supplemental Figures and Tables:

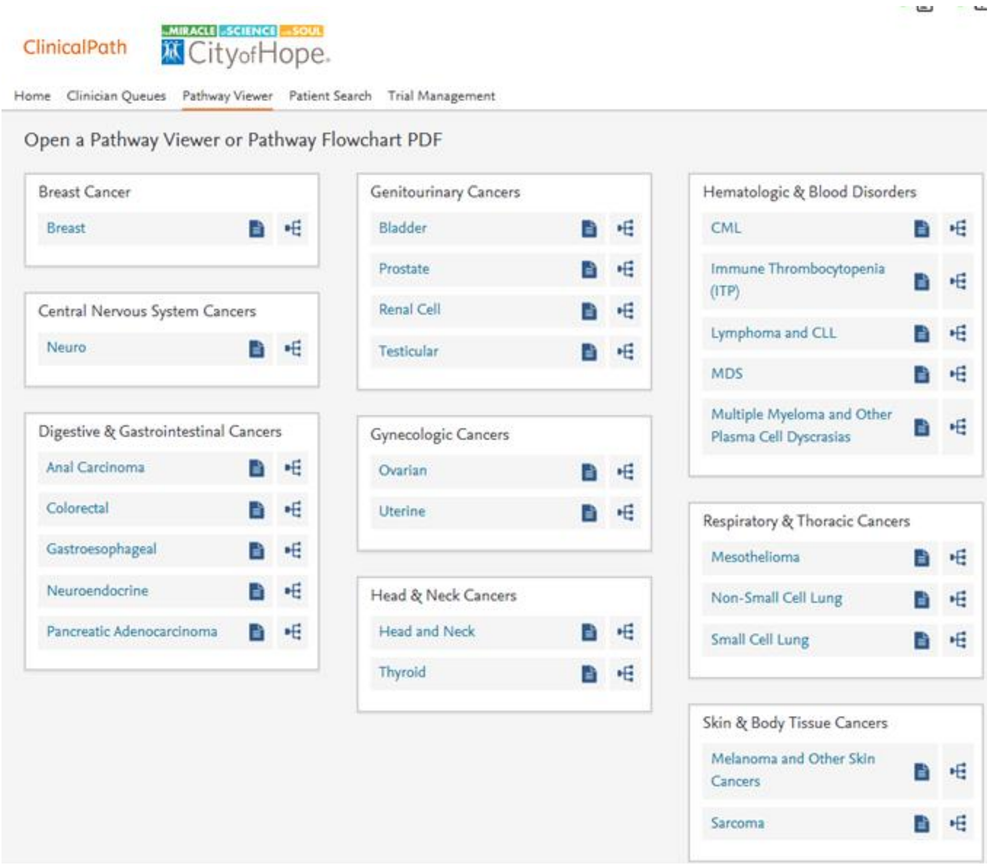

Figure S1: Tumor Types by Disease Categories in City of Hope's ClinPath Pathways.

Table S1: Tumor Board Quality Improvement Questionnaire

Case Submission	
1	Date
2	Submitting Provider
3	Medical Record Number
4	Primary Site
5	Case Description
6	Initial Presentation or Recurrent
7	Histology
8	TNM Stage
9	Question for Radiology
10	Question for Pathology
11	Question for Surgery
12	Question for Medical Oncology
13	Question for Radiation Oncology
14	Question for Genetics
15	Other Questions
16	Question for Tumor Board
Case Discussion Results	

1	Tumor Board Date
2	Medical Record Number
3	Radiology Recommendations
4	Pathology Recommendations
5	Surgery Recommendations
6	Medical Oncology Recommendations
7	Radiation Oncology Recommendations
8	Genetics Recommendations
9	Other Recommendations
10	Overall Tumor Board Recommendations
